# Conscious active inference II: Quantum orchestrated objective reduction among intraneuronal microtubules naturally accounts for discrete perceptual cycles

**DOI:** 10.1016/j.csbj.2025.09.016

**Published:** 2025-09-13

**Authors:** Michael C. Wiest, Arjan Singh Puniani

**Affiliations:** a Neuroscience Department Wellesley College, 21 Wellesley College Rd., Wellesley, MA 02481, USA; b University of Pittsburgh Rehab Neural Engineering Labs, 1622 Locust St, 4th Floor, Pittsburgh, PA 15219, USA

**Keywords:** Microtubules, Orch OR, Predictive coding, Active inference, Consciousness, Optimal control, Bayesian brain

## Abstract

In the first of two companion papers, we argued that classical neural mechanisms proposed to implement conscious active inference had failed to establish their biological plausibility in terms of realistic biophysical models. We further explained that conscious (temporally deep) active inference is mathematically equivalent to the path integral that underlies quantum dynamics. As such, we proposed that a quantum model provides a natural, biologically plausible mechanistic implementation of the processing required by active inference.

In this second paper we review the evidence establishing discrete non-overlapping cycles of perceptual inference, and argue that classical process models have so far failed to motivate or describe these discrete cycles in terms of realistic neural mechanisms. We then point out that the Orchestrated Objective Reduction (Orch OR) theory of consciousness naturally solves this fundamental problem.

Along the way, we review independent strong theoretical and experimental evidence from my (Wiest) lab and others’ supporting the Orch OR quantum theory of consciousness as a collective quantum property of intraneuronal microtubules (MTs). This includes demonstration of room-temperature quantum effects in MTs, MT resonances controlling membrane spiking in living neurons, evidence that volatile anesthetics target MTs to cause unconsciousness, and direct biophysical evidence of a macroscopic entangled state in the living human brain. Intraneuronal MTs thus offer a biologically specific and experimentally supported substrate for implementing conscious active inference in brains.

## Graphical Abstract

**Figure:**
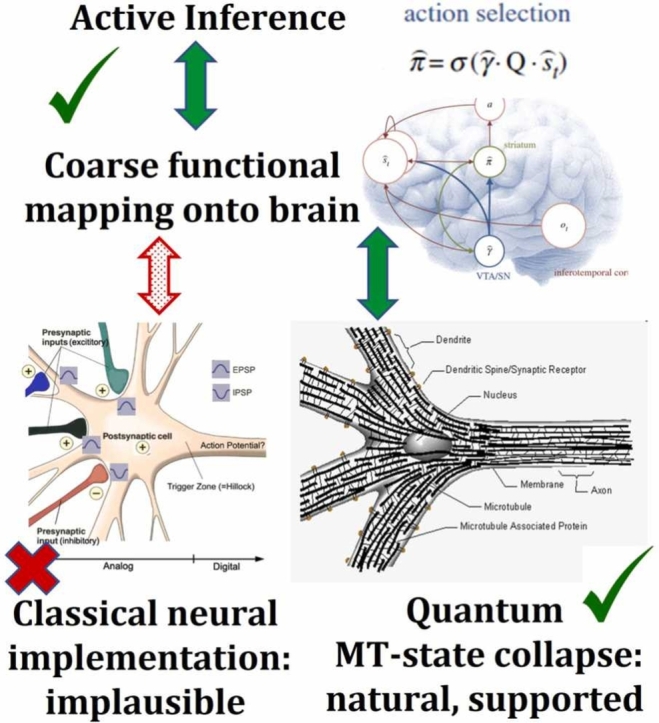

PAPER 2 Contents:

1. The problem of discrete perceptual cycles.
2. Evidence for consciousness as Orchestrated Objective Reduction in brain microtubules.
3. Quantum Orch OR accounts for discrete perceptual cycles.
4. Summary and Outlook: testing and refining the quantum active inference model.

## 1 The problem of discrete perceptual cycles

### 1.1 Consciousness comes in discrete finite-duration moments

In the companion paper (conscious active inference I, Section 3), computational considerations led us to consider irreversible, discontinuous, and temporally indivisible neural dynamics for plausibly implementing active inference. In this Section we point out that the neuroscience of consciousness leads to the same conclusion. Instead of a continuous “stream,” conscious experience is broken into discrete moments of perceptual decision [159], [160], [30], [47], [51], [99]. Each moment represents integration over a short duration followed by a discrete (discontinuous) perception. In particular, each moment of experience somehow integrates a brief duration into the “specious present.” If this were not so we could not perceive movement.

Recall from 2, 3 of the previous paper that temporally deep (conscious) active inference *requires* a period of integration to produce a perceptual or motor output. If two such periods were to overlap in time, they might produce conflicting perceptual interpretations and motor outputs. In active inference it probably doesn’t make sense for the windows of perceptual integration to be overlapping or continuous.

Recall also that our focus on active inference, as opposed to other models of consciousness or perceptual inference, is based on the unique mathematical equivalence that we demonstrated in the previous paper between conscious active inference and the quantum path integral that governs all quantum dynamics. This mathematical equivalence doesn’t apply to any other model of optimized perception and behavior. It rigorously justifies our proposal that a quantum system could *implement* conscious active inference.

How do we know that conscious experience is broken into discrete moments? First, neural signals representing auditory and visual inputs are processed at different latencies, but perceived as simultaneous. This problem of synchronizing signals in conscious perception which are asynchronous in the brain appears to imply that neural signals are integrated over a finite time window to generate the conscious impression of synchrony or serial order between any two stimuli.

This conceptual argument is strongly supported by direct experimental evidence of a minimal duration of neural activation required for conscious perception. By electrically stimulating the cortex of awake humans, Benjamin Libet found that conscious tactile perception required a minimum duration of neural activation on the order of 100–200 ms [106], [107], [108], [109], [119].

Consistent with these results, a large literature on backward masking shows that perception of brief stimuli can be altered or prevented from occurring by introducing new stimuli within a few hundred milliseconds [113], [114], [115], [142]. Similarly, the color phi effect [98] and flash-lag illusion [37], [38] demonstrate that each moment of perception is based on integrating over a few hundred milliseconds of the recent past. A recent comprehensive review catalogs many other fascinating perceptual experiments conclusively establishing that perception comes in discrete non-overlapping temporal windows [47].

**Fig. 1:**
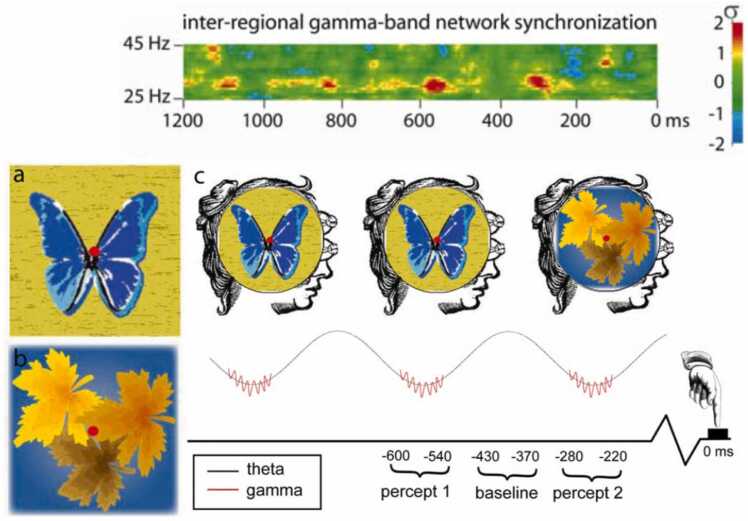
**Discrete moments of experience correspond to rhythmic bursts of large-scale gamma synchronization.***Top*: EEG data demonstrating rhythmic gamma synchronization time-locked to button-presses signaling perceptual switches during binocular rivalry. *Bottom panels*: Schematic depicting left and right eye stimuli during binocular rivalry; and the interpretation of the gamma synchronization data in terms of discrete moments of perception, recurring at a frequency in the theta range. Figures reproduced from Doesburg et al. (2009). Rhythms of consciousness: Binocular rivalry reveals large-scale oscillatory network dynamics mediating visual perception. *PLOS ONE*, 4(7), e6142. https://doi.org/10.1371/journal.pone.0006142, licensed under CC BY 3.0.

An EEG study of binocular rivalry [174] showed that these finite duration conscious moments correspond to bursts of gamma-frequency inter-areal synchronization, which recur rhythmically at frequencies in the theta or alpha range (Fig. 1). Each moment synchronizes activity across distributed areas housing neurons encoding the contents of consciousness at that moment. Experimental results like these ([49], [50], 2006; [99], [139]) support James’ conclusion, argued at length in *Principles of Psychology* [83], that “every cognition is due to one integral pulse of thought.”

This is also true under the active inference formalism: each temporally deep inference about what to do next requires integration over the recent past and possible futures, to decide on one consistent—i.e., “integral”—course of action [127]. In other words, conscious active inference *implies* discrete finite-duration windows of integration [127]. We noted this above and in 2, 3 of the prior paper (conscious active inference I).

It is critical to appreciate that this requirement for temporally deep agents to integrate over finite-duration time windows remains equally true whether we adopt a discrete time formulation or a continuous time formulation of active inference. This is because the sentient agent in active inference optimizes *expected* free energy, which is free energy integrated over some time window into the future [127].

White [167] argued for a “sliding-window” continuous alternative to discrete perceptual update cycles, but this is ruled out by the microstimulation and backward masking effects we reviewed above. That is, under a slide-window picture, new stimuli should be perceived immediately (i.e., after conduction delays) regardless of what stimuli come after them. Instead, a later stimulus can prevent an earlier stimulus from being perceived. The sliding-window hypothesis cannot account for the enormous psychophysical literature on such backward masking [47]. It also appears unable to account for how asynchronous neural signals can be experienced as simultaneous, since new stimuli are supposed to be processed and experienced continuously, but their neural representations are admittedly asynchronous due to unequal conduction delays in different modules and modalities [11], [170].

Fekete et al., [43] articulated a different argument against discrete perceptual updates. First, as we reviewed in Section 3 of the previous article (conscious active inference I), their perspective argued that continuous-time dynamical attractor systems with time constants of the magnitude associated with neuronal population dynamics are simply too slow to serve as the neural implementation. They also argue that continuous dynamical attractors are simply too…*continuous* to implement *discrete* inference updates. “In the ideal scenario this necessitates that the transition between two distinct successive states will be instantaneous…. Thus, regardless of the nature of the proposed states, for the neuronal case for discreteness to be plausible, at least some neurons should switch states nearly instantaneously (i.e., at a rate of change that is qualitatively different from non-switching epochs).” Such near-instantaneous switching among quasi-stable global patterns of brain activity is well-documented ([49], [50], 2006; [99], [139]). The question is: what could its mechanistic basis be?

The classical models are continuous, but both neurophysiological and psychophysical data imply discrete transitions. Remember that continuous *conscious* update models are ruled out by backward masking and microstimulation results implying a discrete update after a minimal duration of neural activation. But the classical models are incurably continuous, even when they explicitly include spikes.

Discontinuous stochastic jumps are postulated in some process models; but, as we reviewed in Section 3 of the companion paper, *at time scales relevant to perception*, those models have no mechanistic basis for the stochasticity they postulate. How do we know this? We saw in the previous paper (conscious active inference I) that neither deterministic nor stochastic process models were able to account for the observed stochasticity of real neurons *in vivo*. The deterministic models could appeal to synaptic failures or input variability, but experimental studies have established that synaptic stochasticity and input variability do not account for the full stochasticity of neurons *in vivo* [19], [21], [45], [8], [82], [9]. Stochastic process models similarly cannot account for the stochasticity they postulate, since real neurons are understood to be governed by the *deterministic* Hodgkin-Huxley equations; and again, neither synaptic stochasticity nor input variability added to the HH model account for the variability of real neurons *in vivo*.

This appears to leave the classical approach without a candidate mechanism to account for the empirically necessary discrete cycles of conscious active inference.

We have raised these points to note that the perceptual cycle of temporally deep active inference is continuous in the classical formulation, without any discontinuity in the physical dynamics to correspond to the cycle of discrete inference/decision/perception events indicated by the evidence we have just reviewed. This issue can be addressed in a classical model by devising a neural threshold for perceptual or behavioral decisions to be “actualized” [147]. A neural threshold is obviously biologically plausible for neurons, which integrate charge and fire a spike if they reach a voltage threshold. But this approach raises the question: what is different about the particular neuron or neurons which ignite a conscious moment when they reach *their* spiking threshold, as compared to all the other neurons who fire spikes unconsciously? The classical model can only address this issue functionally, rather than ontologically, by distinguishing the (experimenter-imputed) *functions* of the neurons whose spikes trigger conscious moments. Active inference process models to date have used idealized rules in which a discrete inferential update is performed at the end of a fixed “theta” or “alpha” rhythm cycles, without any mechanistic basis [124], [125], [3], [54].

Another study showed that inference could be performed in a continuous time model [23] but did not explicitly treat temporally deep (i.e. conscious) agents, who require discrete cycles of planning and inference. As we emphasized above, a continuous formulation without finite-width windows of integration does not describe a conscious agent. “Temporally deep” refers to precisely to this finite duration window of integration. Such integration over simulated future time is evidently necessary to consider and weigh the potential outcomes of various policies (plans) over time.

Parr et al., [126] emphasize the importance of “chunking of time into discrete cognitive units,” or sequences of events, for planning and motor control. They model an agent “inferring when to move” its arm in order to match a rhythmic sensory input. The model relies on an array of idealized neural oscillator “clocks” and infers which clock best explains the sensory input in order to time an appropriate movement. It can account for relatively sudden, “discrete” movements, and it impressively accounts for features of Parkinson’s disease. However, while it captures “proximate” temporal dependencies in the trajectories of distribution parameters, it does not represent whole trajectories in a temporally deep path integral.

More importantly, the discrete movements generated by the model in this context are not spontaneously emerging cycles of perceptual inference. Rather, the study models how an agent can time movements to match predictable external stimuli. Similarly, [52] model spontaneous generation of discrete eyeblinks, but discrete movements are not equivalent to the spontaneously generated non-overlapping discrete cycles of perceptual inference we are trying to account for. Sequences of movements, although they might be describable as discrete events [125], [55], are not sequences of perceptual inference cycles.

None of these models imply discrete non-overlapping cycles of inference in a context with no movement, so they do not represent an account of non-overlapping cycles of perceptual integration and inference in temporally deep agents. For example, in binocular rivalry experiments like those we reviewed above, subjects observe changes in percept even if they are not making movements to report those changes. Perceptual cycles emerge spontaneously even when no movements are demanded by the situation. This is what we need a model to account for.

Fleming, Michel [47] comment: “One might argue that it would be more adaptive for a system to engage in a continuous and dynamic process of perception and planning, with no discrete switches between the two. Further theoretical work will be required to understand whether and how the temporal windowing of perception implied by postdictive effects in perception is adaptive for the control of behavior. One possible advantage is that a slower temporal oscillation of perception and (active) inference allows for the comparison and selection among distinct counterfactual action plans, all conditioned on the same (comparable) world model [81], [91].” This conjecture is consistent with our considerations at the start of this Section.

Rabinovich et al., [139] used rate-based Lotka-Volterra-like models to explore how neural populations could generate sudden discrete transitions in activity patterns. However, these models involved no inferential dynamics: they are not active inference process models. Moreover, like the classical process models we reviewed in the companion paper, these models are not validated by realistic Hodgkin-Huxley (HH) based single-neuron models. HH-based models have not been shown to be capable of robustly behaving like the mathematical idealizations *on fast sub-second time scales*.

Thus, active inference is currently without a demonstrated biologically realistic mechanism to implement discrete perceptual cycles. Recall from the prior companion paper that we *accepted* active inference as a coarse description of how the brain operates. We are not questioning the active inference theory: we are questioning the *implementation* using classical HH-based neurons, because it has not been demonstrated. This applies to the implementation of discrete inferential cycles in the same way it did to the computational operations that we focused on in the prior paper.

## 2 Evidence for consciousness as orchestrated objective reduction in brain microtubules

*The purpose of the present two-part Review,* focused on neural implementations of active inference, is to suggest the collective dipole state of neural microtubules (MTs) as the quantum system used by the brain to implement conscious active inference. Orchestrated Objective Reduction (Orch OR)[134], [69], [70], [71] is the specific quantum microtubule consciousness theory we have in mind. Orch OR identifies moments of conscious experience as a property of the quantum wave-function collapse process, actualizing one out of many possible states of the MT electric dipoles (and associated state of the electromagnetic/photon field). In this context wave-function collapse is roughly synonymous with objective reduction (OR) of the quantum state-vector. This means Orch OR adopts a specific solution to quantum theory’s Measurement Problem, as we will review in Section 3 below.

In non-biological matter OR corresponds to “proto-conscious” events, whereas living creatures have evolved structures like MTs and neurons to “orchestrate” more complex and useful states and dynamics, during which many microscopic processes are integrated into “macroscopic” conscious experiences like ours [168].

In order for this hypothesis to be plausible, we need to establish several dubitable points:

1. That a macroscopic quantum state of neural microtubules can exist in living animals at physiological temperature;
2. The collective MT state must be “programable” by the patterns of electrical activity across the neural membrane, since we know sensory inputs that we experience consciously are encoded in the membrane potential;
3. The MT state must be able to react back on the membrane potential, since we know our behavior is implemented by action potentials in motor neurons; and
4. The collective MT state must span many neurons if it is to incorporate the diverse sensory features that we experience at a given moment, since these are encoded by neurons distributed across cortex: in other words, to solve the phenomenal binding problem.

After reviewing evidence supporting these points, we will be better prepared to appreciate how Orch OR could implement conscious active inference and solve the problem of discrete perceptual cycles, in Section 3 below.

### 2.1 Direct biophysical evidence of a consciousness-related quantum state in living humans

In a recent series of experiments Kerskens and Pérez [137], [93] used a novel quantum entanglement-detection method applied to conscious humans in an MRI scanner. They reported strong evidence for an entangled brain state related to consciousness and working memory performance. They used an unconventional MRI protocol designed to isolate signals from entangled states, and observed an MRI signal that mimicked heartbeat-evoked potentials recorded with EMG. The authors argued the observed signal implied the existence of an entangled brain state that was capable of coupling with the nuclear spins in water molecules that were entrained by the MRI machine. Because the fidelity of the putative spin-entanglement signal correlated with short-term memory performance [137] and the presence or absence of the conscious state itself in sleep vs waking [93], the authors concluded that the quantum brain processes are likely an important part of our cognitive and conscious brain functions. Their interpretation in terms of entanglement has been challenged [165], but that author offered no alternative classical account of the signal they observed. Interestingly, anesthesia research has also implicated nuclear spin in the mechanism of anesthesia [104]. This result is baffling on the assumption that anesthetics work by standard chemical (i.e. electronic) binding at receptors, but is understandable in terms of a quantum model of consciousness [72].

**Fig. 2:**
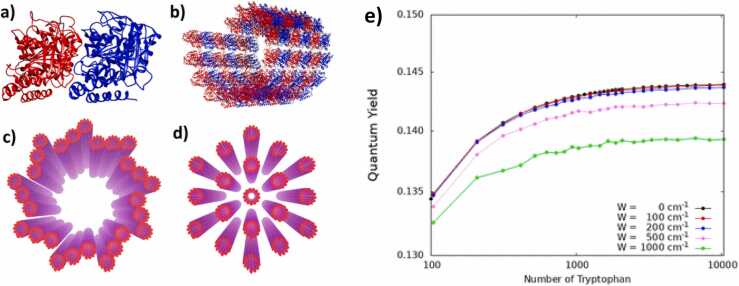
**Microtubule (MT) quantum super radiance at room temperature increases with*****size*****of the MT structure. (a)** Protein structure of a single tubulin dimer, the subunit that polymerizes to form MTs. **(b)** Microtubule segment. **(c)** Centriole formed from nine triplets of MTs. **(d)** Hexagonal bundle of 19 MTs from a typical mammalian axon. **(e)** Strength of room temperature superradiance as a function of the size of a single MT. *Quantum Yield* is a measure of fluorescence intensity: the ratio of the number of emitted photons per time and volume to the number of absorbed photons. *W* parametrizes “structural disorder,” to establish the robustness of the phenomenon. Figures reproduced from Babcock et al. [12], *Journal of Physical Chemistry B*, 128(35), 7941–7953. Licensed under CC BY 4.0. https://doi.org/10.1021/acs.jpcb.4c01991.

Could MTs be the substrate of the consciousness-related quantum brain state? In a word, yes. The hypothesis that macroscopic quantum effects might be functionally relevant for neuroscience has been widely considered physically implausible based on the intuition that the brain is too hot to support delicate quantum effects [155]. Tegmark’s influential estimate was criticized almost immediately on multiple grounds [168], [67]. For example, Tegmark’s analysis modelled a system at thermal equilibrium—which is equivalent to death, and therefore not appropriate for modelling a living system. In any case, we now have decisive experimental evidence that MTs can support collective quantum effects (specifically superradiance) at *room temperature*. Moreover, the strength of the quantum effect increases rather than decreases with increasing size of structures built from MT subunits (Fig. 2).

Superradiance refers to exceptionally bright and coherent emission of light. It is a quantum phenomenon similar to a laser. It is being explored as means of communication and computation through nanofiber waveguides [151], [97], taking advantage of “infinite-range” dipole interactions like those underlying the hypothesized collective MT oscillatory state. This context suggests “the tantalizing possibility that [MT-rich] axons might serve as such waveguides between giant superradiant emitters in the brain” [12]. This is the proffered mechanism for establishing entanglement among MTs in different neurons, necessary for quantum computation and the unity of consciousness.

These results strongly support the possibility that MTs could contribute to the consciousness-related quantum state reported by Kerskens and Perez.

Further evidence for the hypothesis that the biological substrate of consciousness is a collective quantum state of intraneuronal MTs comes from considering the molecular mechanisms by which volatile anesthetics reversibly render us *un*conscious.

### 2.2 Anesthesia, consciousness, and microtubules (MTs)

**Fig. 3:**
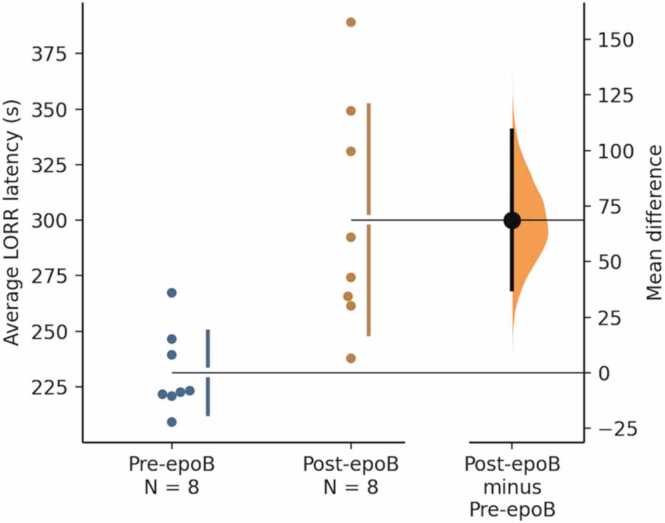
**Rats given a MT-binding drug (epoB 0.75 mg/kg) were resistant to isoflurane anesthesia.** The standard proxy for time to fall unconscious is the latency to Loss of Righting Reflex (LORR), meaning that a rat placed on its back does not right itself. The average LORR latency was 69 s higher in the post-epoB condition as compared with the pre-epoB average, and this difference was statistically significant (two-sided permutation *t*-test; N = 8 rats; p = 0.0016; Cohen’s d = 1.9). The difference remained significant when we omitted eight outliers from the post-epoB dataset (p = 0.005; d = 5.4). Both groups are plotted on the left axes; the mean difference is plotted on a floating axis on the right as a bootstrap sampling distribution. The mean difference is depicted as a dot; the 95 % confidence interval is indicated by the ends of the vertical error bar. Figure reproduced from Khan, S., Huang, Y., Timuçin, D., Bailey, S., Lee, S., Lopes, J., Gaunce, E., Mosberger, J., Zhan, M., Abdelrahman, B., Zeng, X., & Wiest, M.C. [95]. Microtubule-Stabilizer Epothilone B Delays Anesthetic-Induced Unconsciousness in Rats. *eNeuro*, 11(8), ENEURO.0291–24.2024. https://doi.org/10.1523/ENEURO.0291-24.2024, licensed under CC BY 4.0.

My lab recently reported that rats administered with a brain-penetrant MT-binding drug took significantly longer to fall unconscious under the volatile anesthetic isoflurane [95], suggesting that isoflurane causes unconsciousness at least in part by binding to MTs (Fig. 3). The effect size was “large” as assessed by a Cohen’s d value of 1.9. The idea that MT-modulating drugs interact functionally with isoflurane anesthesia was dramatically confirmed in a recent study in mice [105]. These results are consistent with reports that volatile anesthetics bind to MTs [123], [59], [86], and with a behavioral experiment demonstrating that an anthracene-based anesthetic reversibly immobilized tadpoles by acting on their MTs [40]. Similarly, a clinical study of anesthetic usage by human surgery patients given MT-stabilizing chemotherapy (which generally penetrates poorly into the brain) compared to control subjects found a slight anesthetic resistance in the patients given the MT-binding drug [111].

**Fig. 4:**
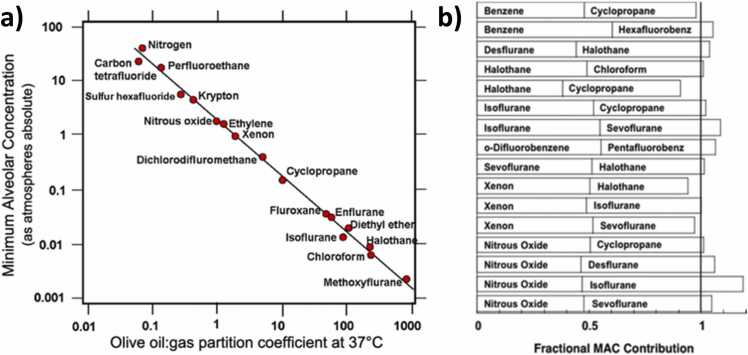
**Anesthetic properties suggesting a common “unitary” molecular target. (a) The Meyer-Overton correlation for inhalational anesthetics.** The effective dose (vertical axis) is predicted by solubility in olive oil (horizontal axis), suggesting a weak physical interaction at an evolutionarily conserved lipophilic target, rather than chemical lock-and-key binding. **(b) Additivity of effective doses (of “MACs”).** One-half the effective dose of one anesthetic plus one half the effective dose of another anesthetic equals one effective dose, even when the two anesthetics have very different effects on a particular ion channel, arguing against ion channels as the primary functional target of inhalational anesthetics. Eger, E.I. II, Raines, D.E., Shafer, S.L., Hemmings, H.C. Jr., & Sonner, J.M. (2008). Is a new paradigm needed to explain how inhaled anesthetics produce immobility? *Anesthesia & Analgesia*, 107(3), 832–848. https://doi.org/10.1213/ane.0b013e318182aedb. Reproduced with permission from Wolters Kluwer Health, Inc. The Creative Commons license does not apply to this content.

Although contemporary authors tend to assume that many molecular targets contribute significantly to causing unconsciousness [116], [74], multiple striking empirical facts appear to suggest that diverse volatile anesthetic compounds act *primarily* on a single highly-conserved molecular target protein to *selectively* abolish consciousness. First there is the venerable Meyer-Overton correlation [90] between anesthetic potency and solubility in olive oil over several orders of magnitude (Fig. 4a). It suggests that anesthetics interact via weak physical interactions such as van der Waals forces among induced dipoles rather than ionic binding. Why? Because the volatile anesthetics are chemically diverse. It appears highly unlikely that they would all fit the same specific chemical binding site on a protein receptor, like keys with different shapes fitting into the same lock. Another alternative interpretation of the Meyer-Overton correlation might be that it reflects the advantage that lipophilic compounds have in penetrating the blood-brain-barrier and lipid-rich brain tissue. If this latter interpretation were true, one would expect more psychoactive drugs to show the same Meyer-Overton correlation. But the correlation is specific to volatile anesthetics.

Similarly, the Meyer-Overton correlation is consistent with the known binding of volatile anesthetics in hydrophobic pockets of diverse proteins [48]. This accounts for the promiscuous *binding* of volatile anesthetics, but fails to account for the specific functional effects on consciousness due to inconsistent effects of binding to the various candidate targets [39].

Moreover, this interpretation (assuming multiple other molecular targets such as ion channels and synaptic receptors) ignores the detailed biophysical modelling of [29](reviewed briefly below) which strongly supports the physical interaction hypothesis, with microtubules in particular (where the functional effects happen in hydrophobic pockets in the tubulin proteins).

Moreover, if the anesthetics target multiple molecular targets, the Meyer-Overton correlation seems to imply that all the targets in diverse proteins share highly similar binding properties. A unitary molecular target might be more plausible than a varying combination of ion channels and other targets.

Second, the effective dose for a given anesthetic varies little across diverse species [39], despite wide variability in the profiles of different ion channels in each animal. A third remarkable property of these compounds is the approximately linear additivity of their effects, despite variable effects of each anesthetic on specific ion channel targets (Fig. 4b). For example, isoflurane activates GABA receptors relatively strongly while cyclopropane has only a small effect—but nevertheless half of an effective dose of isoflurane plus half the effective dose of cyclopropane results in a full effective dose. Fig. 4b shows several such combinations chosen for their differential effects on a particular candidate ion channel. Examples like these appear to show that no single ion channel can account for the unconsciousness caused by inhalational anesthetics. Importantly, a systematic analysis of available evidence also appeared to show that no *combination* of ion channel targets can account for the pattern of empirical results either [39], leaving those authors perplexed. Since then the field has tended to assume that anesthesia is mediated by a combination of ion channels and other targets [116], [74]; but this approach fails to account for the Meyer-Overton correlation and other remarkable facts we just reviewed.

However, none of these analyses ruled out microtubules (MTs) as the primary molecular mediator of inhalational anesthesia. Remarkably, a detailed quantum chemical modeling study found that the potencies of several volatile anesthetics were predicted by their binding affinity to delocalized electron sites within the tubulin subunits that make up MTs [28], [29]. *These theoretical results essentially reproduce the Meyer-Overton correlation by assuming that inhalational anesthesia is primarily mediated by MTs.* This has not been demonstrated for any other candidate molecular target. Thus, MTs could be the primary molecular target which mediates the unconsciousness caused by inhalational anesthetics.

Their promiscuous binding at hydrophobic pockets in a variety of proteins in the brain and spinal cord, is one reason inhalational anesthetics are generally believed to cause unconsciousness by acting on some *combination* of ion channels and receptors, synaptic proteins and gap junctions, mitochondria, and cytoskeletal proteins including microtubules [116], [74], [92]. Moreover, it is likely that other molecular targets besides MTs contribute to anesthesia. For example, binding to GABA receptors does appear to contribute to unconsciousness caused by isoflurane, because mice with non-functional GABARs are somewhat resistant to isoflurane [152]. To a first approximation, if binding to MTs can explain the Meyer-Overton correlation between anesthetic potency and binding at a specific lipophilic site [29], then contributions from other mechanisms would represent the deviations from the linear relation visible in Fig. 4a. It is conceivable that a classical model of consciousness could account for the contribution of MT-binding to causing unconsciousness, but this interpretation is not favored by current evidence [168], [173], [75]. Instead, quantum optical effects in MTs have been shown to be dampened by inhalational anesthetics [87], supporting the quantum view.

So far we have addressed the first dubitable point in our list of four above: we have direct biophysical evidence of a consciousness-related quantum state in the human brain, and we have strong and varied experimental and theoretical evidence that a collective quantum vibration of MTs could be its physical substrate.

Points 2 and 3 from our list above require two-way communication between the collective quantum MT state and voltage dynamics (spikes) on the neural membrane. MTs are well-situated in neurons to integrate electrical activity on the neural membrane (for example via calcium influx during neural activity) and in turn modulate neural membrane voltages and spiking activity (for example by modulating synaptic release probabilities, which are known to be stochastic, “noisy,” or “unreliable”). As such they appear to offer an ideal candidate medium for intracellular integration and adaptive responding. They are evolutionarily plausible candidates because they are present in all eukaryotic cells, in which they perform numerous cellular functions related to integration of environmental signals and coordinating movement [122], [161], [162], [2], [24], [4], [5], [6], [7], [88].

### 2.3 Microtubule resonances span multiple neurons and control neural spiking activity

But we need not speculate further because we have amazing experimental evidence from Anirban Bandyopadhyay and colleagues, that MT resonances predict and control the spiking activity of neurons. They stimulated MT resonances in cultured neurons and observed the resonance state *spanning across multiple neurons* and *controlling membrane voltage* [141], [149], [150]. These experiments strongly support the physical plausibility of the quantum microtubule consciousness hypothesis.

They also address the fourth and final, crucial point in our list above: the necessity for the quantum substrate of consciousness to span multiple neurons. We require this because it is clear that the neural representations of various consciously perceived sensory features are widely distributed across cortex. If the substrate of our experience is the collective state of intraneuronal MTs, it must have simultaneous access to the perceptual representations distributed across cortex, and it must somehow fuse them into a single unified percept. Such a fusion is illusory and epiphenomenal in a classical model [96], but causally efficacious in a quantum model [168].

These results are worth dwelling on briefly because they appear to conflict with Hodgkin and Huxley’s heroic discoveries [76], [77], [78], [79], [80]. How do we reconcile our knowledge that neurons fire deterministically based on whether current inputs drive the membrane voltage to threshold or not (supported by countless experimental studies), with these experimental results in living neurons demonstrating *another way* of controlling a neuron’s spiking activity? In the last century, the field appeared justified in neglecting all but the “skin” of neurons, because Hodgkin and Huxley’s experiments demonstrated that neurons (axons) were capable of producing action potentials even when they had been “hollowed out” [13], [14], [15], [16]. Now it appears that we were reckless in forgetting that the interior might still have functional relevance in the real physiological context [65].

## 3 Quantum orchestrated objective reduction accounts for discrete perceptual cycles

Returning to the problem of discrete non-overlapping perceptual cycles: identifying discrete moments of inference and consciousness with individual quantum collapses of the MT state as described in Orch OR automatically introduces the empirically-supported discontinuity into the dynamics, and provides a physical basis for discrete decision cycles required by active inference and confirmed experimentally. Orch OR also provides an *objective, ontological* basis for distinguishing the conscious brains states from unconscious ones. State-vector reduction violates the continuous “unitary” quantum evolution (such as is described by the Schrödinger equation) to introduce a discontinuous, holistic, irreversible decision. It is actually instantaneous [70] as in the ideal case of [43]. This is a fundamental difference between a continuous local classical model and a quantum physical model incorporating OR.

Recall the perceptual inference cycle that we outlined in Section 2 of the companion paper (active inference I). In step 1 a prediction is generated from prior beliefs about the world including the agent itself, and in step 2 the prediction is compared to new inputs to calculate updated, corrected posterior beliefs. These posterior beliefs then act as the priors to generate predictions for the next cycle of inference. We further outlined a rough match between cortical circuitry (Fig. 1 of the companion paper) and neural modules, and variables in active inference process models (Fig. 2 of the companion paper). This coarse match suggested the computations that neurons in the different modules contribute to the overall predictive processing algorithm. In turning from a classical to a quantum implementation, we *retain* the functional mapping suggested by active inference process models. The neurons retain their hypothesized functional profiles, but the required stochasticity and path integration is provided by the quantum Orch OR dynamics.

**Fig. 5:**
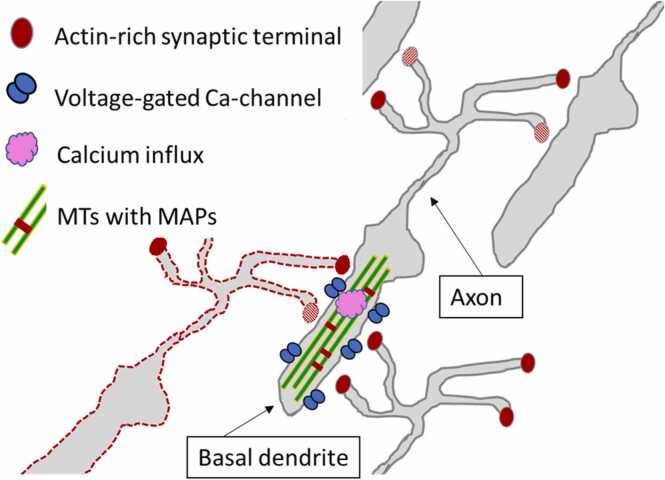
**Schematic implementation of two-way communication between membrane voltage and collective quantum MT state.** An active neuron (*red dashed outline*) excites the postsynaptic membrane causing calcium influx (*pink cloud*) through voltage-gated channels (*blue pairs*), which modulates the state of MTs (*long green bars*) and MAPs (*short red bars*). This is how sensory information may be imprinted onto the MTs state. After the optimizing dynamic of the quantum path integral, objective reduction (OR) of the collective MTs state occurs, generating a sensory-updated “posterior” state. This state acts back on the membrane spiking activity through light-sensitive actin at the synapses (*red ovals*) acting as “logic gates.” MTs in the axon, acting as waveguides to establish the quantum entanglement across many neurons necessary for quantum computation and phenomenal binding, are not shown for clarity.

Under the Orch OR picture (Fig. 5), the two steps of the inference cycle would play out as follows:

• In Step 1: the information encoded in the predictive neural populations is transmitted to the collective state of MTs inside those neurons, for example via activity-dependent calcium influx through voltage-gated calcium channels which are ubiquitous in cortical dendrites. At the same time, sensory information representing current observable outcomes is likewise imprinted into the vibrational state of intraneuronal MTs in sensory areas.
• In Step 2: Network interactions allow the fusion of the predictive and outcome representations from Step 1 into a collective MT state of the combined populations. Classically impossible quantum integration of these MT states allows the quantum evolution to find a next state that minimizes the physical action, which has been orchestrated (by the neural activity pattern) to represent the agent’s prior beliefs and the corrections due to sensory inputs. Due to this orchestrated correspondence, minimizing the physical action can be interpreted as minimizing the “expected free energy” of active inference, which practically means *optimizing* our beliefs and actions. The “next state” is *actualized,* or *realized,* upon objective collapse of the MT state-vector representing superpositions of possible beliefs. In other words, Orch OR of the collective MT state generates the sensory-updated posterior belief-state, which is transmitted back to influence voltage spiking activity on the membrane (for example by modulating synaptic failure probability), to serve as the prior for the next cycle.

### 3.1 Objective collapse criterion

Recall from the previous paper (conscious active inference I) that quantum theory has a Measurement Problem (MP), in that the theory determines probabilities of measurement outcomes but does not define what physical process counts as a measurement. Objective Reduction theories address the MP by positing that state-vector reduction (wavefunction collapse) is real, objective, physical process. The so-called Diosi-Penrose (DP) objective reduction (OR) scheme [129], [130], [131], [132], [133], [33], [34] assumed in Orch OR is one of a number of OR models in which state collapse is induced by gravity [136], [62], [89].

The intuition is that superpositions of masses in different spatial positions will be unstable because of the difference in gravitational self-energy implied by the different spatial configurations. Under the DP OR scheme, the average time *T* until spontaneous collapse is given by

(1) $$T\approx \frac{h}{2\pi E}$$

where *h* is Planck’s constant and *E* is the gravitational self-energy of the difference between the two superposed distributions of mass (in the simplest case)[70], [71]. In other words, *E* is the energy to move the masses through the gravitational field (spacetime geometry in general relativity) from one superposed configuration to the other. The average collapse time is analogous to the half-life of a radioactive decay. A larger difference in energy implies a shorter collapse time. In order for Orch OR to be viable as a theory of consciousness, the quantum system that is to implement Orch OR must remain sufficiently shielded from environmental decoherence.

To apply this collapse theory to the MT system, Hameroff and Penrose [70] considered superpositions of different conformational states of tubulin dimers within a MT. They assumed conformational displacements of these dimer subunits by *d* = 0.2 nm, about one tenth the diameter *r* of each monomer making up a tubulin dimer. They modeled spatial displacements of the tubulin dimers at three different levels: (i) as partially separated protein spheres, (ii) as completely separated atomic nuclei, or (iii) as completely separated nucleons (protons and neutrons).

For case (i), overlapping monomer protein spheres, they derived the energy *E* of partial separation as

$$E=\;\frac{GM^{2}d^{2}}{2r^{2}}(1-\frac{3d}{8r}+\frac{d^{2}}{80r^{3}})$$

where *G* is the gravitational constant, *M* is the monomer mass of 55 kDaltons, *r* is the radius of a monomer sphere, and *d* = *r*/10 is the superposition separation.

Case (ii), describing tubulin as two arrays of carbon atoms, results in a simpler formula because the carbon nucleus displacement is greater than its radius (complete separation):

$$E\approx \;\frac{Gm^{2}}{a_{c}}$$

where *m* is the mass of a carbon atom (12 Daltons) and $a_{c}$ is the carbon nucleus radius. Case (iii) results in the same approximate formula with *m* and $a_{c}$ replaced by the nucleon mass and radius. These displacement energies are for single atoms or nucleons, respectively, so they are multiplied by the number of elements per tubulin to give single-tubulin displacement energy estimates.

The latter two cases result in the largest energy estimates, which dominate the collapse time according to Eq. 1 above. Given these single-tubulin estimates of the displacement energy that determines collapse time, [70] estimate the number of tubulin dimers required to be combined in a coherent collective superposition in order to give collapse times on the order of 500 ms—the time scale of the discrete perceptual moments we want to account for.

On this basis they estimate that of order 10^9^ tubulins must be in superposition collectively to achieve collapse times of order 500 ms. Given roughly 10^7^ tubulin per neuron [169], and assuming 10 % of them participate in the collective state, corresponds to a population of about 1000 neurons. Shorter collapse times would result from larger populations in superposition.

The picture sketched above is the original proposal [67], [70]. Since then, Hameroff and Penrose have raised the possibility that longer perceptual moments could emerge as slow “beat frequencies” generated by the interaction of superposed states that individually have much faster, but slightly differing, collapse rates. This model is analyzed in [71].

Thus, although there are likely unknown relevant factors not accounted for in these simple calculations, we see that Orch OR provides an objective criterion that generates discrete non-overlapping window of integration and collapse that correspond directly to cycles of perceptual inference in active inference. That means the theory provides an objective criterion for the end of a perceptual inference cycle specified in terms of physical parameters of the system.

In the first paper (conscious active inference I) we reviewed the precise mathematical equivalence between conscious active inference and the quantum path integral that expresses how probability amplitudes evolve during the time between measurements—measurements that we are now understanding as objective reduction (OR) rather than merely interacting with some experimenter’s apparatus. The DP OR collapse criterion determines how long that interval between collapses lasts each time based on the current physical state. After the path integral computes probability amplitudes (beliefs) that optimize the action (expected free energy), the state collapse implements the selection of an inferred optimal perceptual interpretation and behavior response.

This is the proffered solution to the problem of spontaneously occurring, non-overlapping discrete cycles of perceptual inference in conscious active inference, under Orch OR.

If the Penrose OR scheme is ruled out by future experiments, there are other principled OR theories we can consider [118], [128], [135], [136], [62], [63], [89].

*

How would Orch OR implement the operations in Table 1 of the companion paper? Given the quantum MT system able to represent the current situation and possible future plans, the quantum path integral can be interpreted as performing exact inference with no variational approximation nor any need for local gradient descent (previous paper, Table 1 row a). The quantum dynamic finds a global optimum. The stochasticity is intrinsic quantum indeterminacy (row i). There is no need for a mean-field approximation (row e) because the quantum dynamic is an indivisible stochastic process. The weighted sums, multiplications, and normalization are performed automatically by the quantum path integral (rows b, f, g). The linearity of the weighted sums, that was problematic for the classical implementation, is intrinsic to quantum dynamics under QM or QFT. Model averaging (row d) might be included in the path integral as well. Readout (row h) would potentially still need to be accomplished by separate downstream neural populations.

**Table 1:** **Problems for a process theory of conscious active inference**, and potential solutions available to classical and quantum process theories.

	**Problem**	**Classical process**	**Quantum reduction process**
a	Approximations	Gradient, mean field	Inference exact, learning approx
b	Probabilistic optimization	Implausible	Automatic
c	Discrete decision moments	Maybe	Automatic
d	Quantum cognition	Maybe	Maybe
e	Memory capacity	Linear in number of units	Exponential
f	Unity of consciousness	Reducible to local interactions	Irreducible
g	Conscious causation	Epiphenomenal	Efficacious, advantageous
h	Encoding/Affect/Valence	Observer-construed, functional	Ontological
i	Nonalgorithmic understand	Implausible	Possible
j	Subject arrow of time	Implausible	Automatic

Recall that we have a rigorous basis for asserting that the quantum system can implement these computations. That rigorous basis is the mathematical *equivalence* of conscious active inference and the quantum path integral that we established in detail in the prior paper. That is why the path integral automatically *implements* all the requisite computations.

If this seems abstract, recall the quantum eye-tracking model that we reviewed in the prior paper [17]. It can be understood as a *process model* illustrating concretely how this algorithm could be incorporated into the brain. It showed how classical neural activities could sculpt the quantum potential governing the evolution of the hypothetical quantum brain process through the Schrödinger equation. The Schrödinger equation governs the evolution of the wave function representing the probability amplitude of making an eye movement to a particular position. In this respect it is equivalent to the quantum description of a single particle moving in a potential field.

Recall also that the Schrödinger equation is a special case of the quantum path integral that is the *general* formulation of quantum physics. That means this model is equivalent to a path integral whose probability amplitudes are squared at each discrete time step to represent the “measurement” outcomes, which are optimal next targets for an eye-movement. That is why we are presenting it as a process model of quantum active inference that takes advantage of the quantum path integral.

Lest this equivalence of the Schrödinger equation to the path integral formulation be dismissed as a mere metaphor, or loose analogy, we will sketch the derivation of the Schrödinger equation for a single particle—whose position may be interpreted as the current target position for an eye movement—from the path integral.

“In the path integral approach, the evolution of a state is given by the transition function *K*(b, a). [See the prior paper, conscious active inference I, for our brief review of the quantum path integral and transition function.] From a classical point of view, this can be viewed as the analogue of Huygens’ principle, where the evolution of a wave can be determined by assuming that each point along a wave front emits a new wave front. The integration over all these infinitesimal wave fronts then gives us the overall evolution of the wave front. Mathematically, this is given by:” [85]

ψ(x_j_, t_j_) = ∫ K(x_j_, t_j_; x_i_, t_i_) · ψ(x_i_, t_i_) · dx_i_

integrated from minus to plus infinity. Here *ψ* is a wavefunction representing the probability amplitude for finding the particle at x at time t. Given the non-relativistic classical action in the absence of any potential (which is the kinetic energy),

$$S\;=\;\int \;\frac{m{v_{i}}^{2}}{2}\cdot \mathrm{dt}$$

the path integral over e^i*S*^ [85] gives the non-relativistic transition function from position (or state) a to state b:

$$K(b,a)\;=\;(m/2\pi {\left(t_{b}-t_{a}\right))}^{1/2}\cdot \exp(\;\frac{\mathrm{im}{\left(x_{b}-x_{a}\right)}^{2}}{2\left(t_{b}-t_{a}\right)}).$$

Based on this transition function, the time evolution of the wavefunction from *t* to *t + ε* is given by:

$$\psi (x,\;t\;+\varepsilon )\;=\;\int \;(m/2\pi {\left(t_{b}-t_{a}\right))}^{1/2}\cdot \exp(\;\frac{\mathrm{im}{\left(x-y\right)}^{2}}{2\varepsilon })\cdot \psi (y,\;t)\cdot \mathrm{dy}$$

To perform the integration, one substitutes *dη* for *dy*, where *η = y – x*, and then Taylor expands the left-hand side in terms of *t*, and the right-hand side in terms of *η*. Since the higher order terms in *ε* vanish in the limit *ε* →0, we keep only the linear terms in *ε.*

$$\psi (x,\;t)\;+\;+\varepsilon \frac{\partial \psi }{\partial t}\;=\;\int \;(m/2\pi {\left(t_{b}-t_{a}\right))}^{1/2}\cdot \exp(\;\frac{\mathrm{im}\eta^{2}}{2\varepsilon })\cdot (\psi (x,\;t)\;+\eta \frac{\partial \psi }{\partial x}+\frac{1}{2}\eta^{2}\frac{\partial^{2}\psi }{\partial x^{2}}+\ldots )\cdot d\eta$$

The first term in the integral reduces to *ψ*(*x, t*), and the linear term in *η* inside the integral goes to zero because it is linear, leaving:

$$i\frac{\partial \psi }{\partial t}\;=\;\frac{-1}{2m}\frac{\partial^{2}\psi }{\partial x^{2}}$$

which is the Schrödinger equation for a free particle. It is straightforward to include a potential in the path integral to derive the Schrödinger equation for a particle in a potential, the basis of the quantum eye-tracking model we highlighted above.

Thus we see that a system evolving according to the Schrödinger equation instantiates the quantum path integral, which we showed in the companion paper is mathematically equivalent to temporally-deep (i.e., conscious) active inference.

It is critical to appreciate, however, that the path integral computes probability amplitudes for outcomes which are not realized until a “measurement” is made. In the Behera et al. model [17], these measurements occurred implicitly at each time step upon squaring the complex wavefunction to obtain a probability distribution from which to select the next actualized (or inferred, under active inference) target location. This arbitrary implementation of a “measurement” at each discrete time step in the quantum eye-tracking model, without any collapse mechanism or theory of measurement, is analogous to the inference steps added “by hand,” without mechanism, at the end of each cycle of a theta rhythm in temporally deep classical process models like [124], [53].

The mechanism that replaces these *ad hoc* schemes under our proposal is Orchestrated Objective Reduction (Orch OR) of the collective MT state which has been “programmed” by the pattern of spiking activity across the neural membranes, analogously to the quantum potential in the quantum eye-tracking model [17]. In this way, arbitrarily enforced windows of integration and inference are replaced by time steps whose duration is determined dynamically by the size of the current coherent superposed MT state, according to the objective criterion specified in Eq. 1.

This gives the brain a mechanism for adapting its pre-conscious integration time to different behavioral contexts. Contexts requiring more frequent inferential updates could be met with recruitment of larger neural populations (or a larger fraction of entangled MTs); while situations demanding longer integration of sensory evidence to reach a reliable inference could be met by isolating smaller coherent populations, resulting in longer times until collapse.

Fig. 5 schematically illustrates a biologically plausible way to operationalize this picture at the cellular level. In Fig. 5, the neuron outlined in *red dashes* has just fired an action potential, resulting in postsynaptic calcium influx (pink cloud) through voltage-gated calcium channels in the dendrite (*blue pair*). MTs themselves and MT-associated proteins (MAPs) are both functionally sensitive to intracellular calcium [143], [18], [36], so their state is plausibly modulated by the calcium influx from membrane electrical activity in the recent past.

We suggest the metaphor of a guitar, in which the vibrating MTs are the strings, the MAPS are frets that constrain allowable frequencies, and the calcium is a finger strumming strings. Different arrangements of MAPs and different spatial patterns of membrane activity will excite the MTs into distinct “chords” resonating across the whole MT orchestra, as it were. This is how the current neural code may be imprinted onto the collective MTs state, so that it represents the current situation and possible future plans.

By hypothesis, this state evolves according to the quantum path integral dynamic until an objective criterion specified in Orch OR is met, at which time OR occurs and an updated “posterior” conscious percept is generated.

As noted above, in order for this new collective state of the intraneural MTs to influence behavior, it must be able to react back on the membrane voltage. Why? Because our behavior is generated by activating muscles, which require action potentials in motor neurons. We have experimental evidence from the Bandyopadhyay lab (reviewed above) directly supporting that MT resonances do in fact control membrane voltage including spiking activity, but how can we envision this influence concretely?

Well, another major component of the cytoskeleton are actin polymers, which cooperate with MTs in single-celled organisms to control movement [35]. In neurons actin is enriched at synapses, and is responsible for the literal movement of dendritic spines (the sites of synaptic contacts) in neurons [112], [46] and complex regulatory functions in presynaptic terminals [60]. In addition, actin can exist in a liquid crystal state [154], [166], [58]. Liquid crystal materials are used in optical computers as programmable logic gates [171], [25], [26], [42], [94]. Given that MTs have specific arrangements relative to synapses, are superradiant and predicted to act as waveguides analogous to nanowires in optical computers, it is natural to speculate that the collective MTs state may modulate neural activities by modulating synaptic transmission through the interaction of the photon field with polarization-sensitive actin at the synapse. In turn the modified light or actin itself could modulate synaptic vesicle release machinery in presynaptic terminals.

An advantage of this picture is that it does not violate the Hodgkin-Huxley dynamics believed to govern membrane voltage. Rather, the spiking activity is modulated via the known “unreliability” or “noisiness” of synaptic transmission in the central nervous system in particular. Thus, this cartoon, though speculative, is consistent with known neurophysiology in a way that classical models of active inference arguably are not.

### 3.2 Interpretations of quantum theory

As we noted in the previous paper (conscious active inference I, Section 4), quantum theory relies on the concept of a measurement in order to produce meaningful predictions, but provides no definition of what physical interaction or process actually constitutes a “measurement.” A review of approaches to this measurement problem (MP) may be found in [164].

Approaches to the MP may be broadly classified into so-called “psi-epistemic” and “psi-ontic” interpretations or theories. The former take the wavefunction (or quantum state) to partially represent an agent’s knowledge about the system being measured, while the latter understand the wavefunction to represent an objective physical state. One class of psi-epistemic theories is known as Quantum Bayesianism, updated to Qbism for short [31], [56], [57].

The discontinuous change upon measurement suggests the epistemic interpretation, but a range of psi-epistemic views were shown to be inconsistent with standard QM by Pusey, Barret, and Rudolph (PBR)[101], [102], [138]. In some cases, psi-epistemic theories make empirically distinct predictions from standard QM. Experiments aimed at this issue have so far supported standard psi-ontic quantum theory and failed to support a range of psi-epistemic variations [121], [140].

The PBR theorem assumes there is some ontological part of the quantum state even in psi-epistemic theories. Because Qbism is more “radically subjectivist” than this, it evades the PBR theorem. However, Qbism appears to lack standard QP’s power to explain basic physical phenomena such the solidity of objects and properties like electrical conductivity [156], [22], [73], so it does not represent a fully developed alternative to standard QM. The quantum active inference formalism of Fields, Friston et al. is a principled quantum Bayesian theory [44] developed from standard QM rather than from a speculative epistemic revision of standard QM.

However these issues may be resolved, we can say unequivocally that our proposal does not work unless OR is true, and epistemic quantum theories are false. We have therefore adopted the objective reduction (OR) interpretation, which views the wavefunction as an objective physical state and its collapse upon measurement as an objective physical process. Different theoretical frameworks predict different specific dynamics of this physical “measurement” process by which nature selects actual physical outcomes from its list of possibilities.

### 3.3 The quantum memory advantage

Aside from accounting for the empirically discrete and irreversible nature of the “stream” of consciousness, identifying moments of experience with Orch OR events also opens up the potential evolutionary advantages of quantum computing. This is because quantum computing depends on state collapse to produce useable outputs. Although active inference prescribes optimal actions, these actions are of course limited by the agent’s physical and computational resources [103], [61]. A quantum substrate embodying Orch OR represents huge potential advantages over a classical brain—quantum computational advantages including quantum associative memory advantages [146], [163], [32]—while at the same time accounting for how that complex physical state distributed across the cortex could correspond to a unified experience [168].

In particular, combining Grover’s quantum search algorithm with a neural network architecture leads to polynomial or even exponential increase in *memory capacity* as compared to classical neural network models of associative memory [1], [10], [144], [145], [146], [157], [158], [163], [172], [32]. The quantum memory outperforms its classical counterpart because it eliminates the interference, aka *cross-talk,* among different memory patterns that plagues classical associative memory networks [32].

This is a significant issue for neuroscience. Although human memory is known to be highly fallible and subject to confabulation, especially in a courtroom context [100], controlled experimental testing has established that humans have immense memory capacity [153], [20]. Moreover, contrary to the general assumption that our memories are highly compressed abstract representations, [20] specifically showed that our “massive” capacity for images comprises sensory *details*, not just compressed abstract semantic representations.

The challenge for classical neuroscience is that capacity in classical neural network models of associative memory like the Hopfield model grows only *linearly* or sublinearly with the number of neural units [117]. Consistent with the Orch OR account, the cytoskeleton does play a major role in memory [27]. It virtually goes without saying that memory capacity is one of the inescapable constraints that active inference must operate under [103], [110]. Presumably biological agents like humans store episodic memories in order to use them to make more adaptive decisions in the future.

In the previous paper (conscious active inference I, Section 3), we noted that humans maintain implicit memories of at least 35 syllables during speech perception [41]. Consistency requires us to face the fact that we are actually maintaining specific episodes in memory for *decades,* because of their potential utility in making future real-time judgments and choices—in a single “moment.” We believe it is fair to say that no model—classical or quantum—has yet described how humans can efficiently interface with this “massive” memory [20] so as to make decisions in a single “trial,” informed by a lifetime of experience.

But again, classical memory models are limited to order N memories in a network of N neural units. For example, a classical network of N = 100 neurons might only reliably store 15 memory patterns, and this grows to only 150 memories for N = 1000 neurons. In contrast, in quantum associative memory models the capacity grows as a power of N, or even exponentially [1], [10], [144], [145], [146], [157], [158], [163], [172], [32]. We suggest that the model with the greater memory capacity has the advantage for explaining our amazing ability to base momentary decisions on remembered individual episodes accumulated over decades.

## 4 Summary and outlook: testing and refining the quantum active inference model

### 4.1 Summary

We have argued that realistic classical neural models of predictive processing have failed to establish their biological plausibility, because the required addition and multiplication operations appear to take too long for real-time inference, especially relying on relatively slow and unstable classical attractor mechanisms. For classical stochastic models the required number of samples appears too large to be carried out quickly enough by realistic numbers of classical conductance-based spiking neurons.

This motivated us to consider an alternative quantum implementation, Orchestrated Objective Reduction (Orch OR) of a collective intraneuronal state of microtubules (MTs). We reviewed direct biophysical evidence of a consciousness-related macroscopic quantum state in the human brain, as well as experimental demonstration of room-temperature quantum optical behavior of MTs, and that MT resonances control membrane voltage and neural spiking activity in living neurons. Evidence that volatile anesthetics target MTs to cause unconsciousness further supports the Orch OR picture.

Table 1 presents a comparison of the classical and quantum process theories in terms of specific implementation issues and other theoretical issues. Row b (*shaded orange*), regarding the plausibility of classical mechanistic accounts of probabilistic processing and active inference, was the primary focus of the previous article. The *white rows* indicate issues that we discussed more briefly. The rows *shaded in grey* indicate issues that we only touched on, but which we note here for completeness. The issue of the unity of consciousness (row f) and the problem of evolutionary epiphenomenalism (row g) are discussed in more depth in [168]. The problem of phenomenal affect (row h), aka hedonic value, is addressed from a quantum perspective in [68], and from an active inference perspective in [84].

Given its falsifiability, its theoretical and experimental support, and its ability to account for the unity and evolutionary relevance of consciousness among other merits summarized in Table 1, we conclude that Orch OR is the leading candidate for a fundamental theory of consciousness and sentient active inference. Recognizing MTs as the substrate of consciousness also naturally accounts for the remarkable “continuity between life and consciousness” noted by many authors [148], since MTs underlie integration and coordination functions in evolutionarily ancient single-celled organisms lacking neurons.

### 4.2 Outlook

The functional importance of quantum entanglement in conscious agents has been *derived* from within the active inference formalism itself [44], so we expect more serious investigation of the role of quantum physics in biology and consciousness. The Orch OR hypothesis, defended here, that consciousness is a property of the quantum collapse of a collective state of intraneuronal microtubules, can be further tested by in-vivo brain spectroscopy experiments [120]. That is, MT-related peaks in the brains of waking insects or mammals should be dampened or otherwise altered under anesthesia [29]. These experiments will likely prove technically challenging because the signal must be detected in a large background. Variations of the quantum MRI approach of Kerskens and Perez may provide alternative routes for experimentally testing the theory. Another avenue is suggested by [64], who predict characteristic “scatter” or noise around sinusoidal membrane potential oscillations, that should quantify the “quantum-like” noise in the neuron’s membrane potential fluctuations.

The present proposal of course leaves many unanswered questions for future work. Orch OR may provide for exact inference for perception and real-time motor control, while still making use of Hebbian-like classical gradient-descent plasticity rules for approximately optimal learning over longer time scales (Table 1 row a). Future work aimed at reconciling active inference with the quantum cognition literature may be liberated from the bias that the neural implementation must be a classical dynamical system [66]. There are questions about how the brain may modulate the rate of Orch OR collapse events based on the “urgency” of the current situation, and there is the fundamental question of how specific experiences are encoded in the collective MT state. This latter question may not be as daunting as it appears. Due to the widespread presence of voltage-gated calcium channels in cortical dendrites, the spatiotemporal pattern of spiking activity on the neural membranes is mirrored in the intracellular calcium concentration, which likely imprints this pattern into the collective MT vibrational state. Thus, much of what we have already learned about the *neural code* will likely translate in a straightforward way to the *MT code*.

If and when the field achieves a consensus that the brain basis of conscious active inference is quantum physical in nature, we will have entered a new epoch in our understanding of ourselves as conscious beings in a physical universe. The outlook is: exciting.

## CRediT authorship contribution statement

**Michael C. Wiest:** Writing – review & editing, Writing – original draft, Supervision, Investigation, Conceptualization. **Arjan Singh Puniani:** Writing – review & editing, Investigation.

## Declaration of Competing Interest

The authors declare no competing financial interests.
